# Two new species of yellow-shouldered bats, genus *Sturnira* Gray, 1842 (Chiroptera, Phyllostomidae) from Costa Rica, Panama and western Ecuador

**DOI:** 10.3897/zookeys.402.7228

**Published:** 2014-04-16

**Authors:** Paúl M. Velazco, Bruce D. Patterson

**Affiliations:** 1 Integrative Research Center, Field Museum of Natural History, 1400 S. Lake Shore Drive, Chicago, IL 60605, USA; 2 Division of Paleontology, American Museum of Natural History, Central Park West at 79th St., New York, NY 10024, USA

**Keywords:** Neotropics, Stenodermatinae, *Sturnira bakeri*, *Sturnira burtonlimi*, Systematics, Taxonomy

## Abstract

Two new species of yellow-shouldered bats *Sturnira* Gray, 1842 (Chiroptera, Phyllostomidae) from Central America and western South America are described using molecular and morphological data. The two new species, which occur in Costa Rica and Panama and in western Ecuador, were previously confused with *S. ludovici*, and *S. lilium* and *S. luisi*, respectively. *Sturnira* now includes 22 described species, making it the most speciose genus in the Neotropical family Phyllostomidae.

## Introduction

The genus *Sturnira* Gray, 1842 (Phyllostomidae, Stenodermatinae) includes at least 23 monophyletic clades of frugivorous bats that are all endemic to the Neotropics. Their collective geographic range extends from Mexico and Lesser Antilles to northern Argentina (Velazco and Patterson 2013). The genus includes small to large (10–68 g) bats found primarily in tropical lowland and montane forest from sea level to at least 3,600 m, but the greatest diversity in the genus occurs on the forested slopes of the Andes where at least 11 species occur (Koopman 1978, Gardner 2008, Velazco and Patterson 2013).

Like other phyllostomid genera (i.e., *Carollia* [Solari and Baker 2006, Pacheco et al. 2004, Velazco 2013] and *Platyrrhinus* [Velazco 2005, Velazco and Patterson 2008, Velazco et al. 2010]), the diversity of *Sturnira* has grown substantially from recent revisionary studies (Iudica 2000, McCarthy et al. 2006, Velazco and Patterson 2013). Only 14 species were recognized in *Sturnira* in the last world checklist (Simmons 2005), but since then, three new species have been described, *Sturnira koopmanhilli* McCarthy et al., 2006, *Sturnira perla* Jarrín-V & Kunz, 2011, and *Sturnira sorianoi* Sánchez-Hernández et al., 2005. In addition, a molecular phylogeny of the genus (Velazco and Patterson 2013) uncovered three lineages that do not correspond to any of the species described to date–these were referenced as *Sturnira* new species 1, 2, and 3 (sensu Velazco and Patterson 2013). The first of these new taxa, *Sturnira* new species 1, occurs in Costa Rica and Panama and belongs to a clade that also includes *Sturnira hondurensis*, *Sturnira ludovici*, and *Sturnira oporaphilum*. The second, *Sturnira* new species 2, occurs in western Ecuador and is the sister species of *Sturnira parvidens*. The third, *Sturnira* new species 3, is one of the most widely distributed species in the genus. Found in eastern Ecuador, eastern Peru, Venezuela, Guyana, Suriname, French Guiana, and Trinidad and Tobago, it belongs to a clade that also includes *Sturnira angeli*, *Sturnira luisi*, and *Sturnira paulsoni* and has long been confused with *Sturnira lilium*. Here we describe two–*Sturnira* new species 1 and 2–of the three new species uncovered by the analyses of Velazco and Patterson (2013).

## Methods

Guided by the phylogenetic studies of Velazco and Patterson (2013), we describe in this report *Sturnira* new species 1 and 2 (sensu Velazco and Patterson 2013), and compare them to the most closely related and sympatric species. External and osteological characters examined were based on, but not restricted to, those defined by Pacheco and Patterson (1992) and Iudica (2000). We follow Miller (1907) in assigning homology for the premolars: 1st upper premolar (P3), 2nd upper premolar (P4), 1st lower premolar (p2), 2nd lower premolar (p4).

The specimens examined by this study and tissues used by the study of Velazco and Patterson (2013) are deposited in the following Recent mammal collections:

AMNH American Museum of Natural History, New York, New York.

CM Carnegie Museum of Natural History, Pittsburgh, Pennsylvania

FMNH Field Museum of Natural History, Chicago, Illinois

LSUMZ Museum of Natural Science, Louisiana State University, Baton Rouge, Louisiana

MSB Museum of Southwestern Biology, University of New Mexico, Albuquerque, New Mexico

MUSM Museo de Historia Natural de la Universidad Nacional Mayor de San Marcos, Lima, Peru

MVZ Museum of Vertebrate Zoology, University of California, Berkeley, California

QCAZ Museo de Zoología of the Pontificia Universidad Católica del Ecuador, Quito, Ecuador

ROM Royal Ontario Museum, Toronto, Ontario, Canada

TTU (TK) Museum of Texas Tech University, Lubbock, Texas

USNM National Museum of Natural History (formerly the U.S. National Museum), Smithsonian Institution, Washington, D.C.

**Figure 1.**
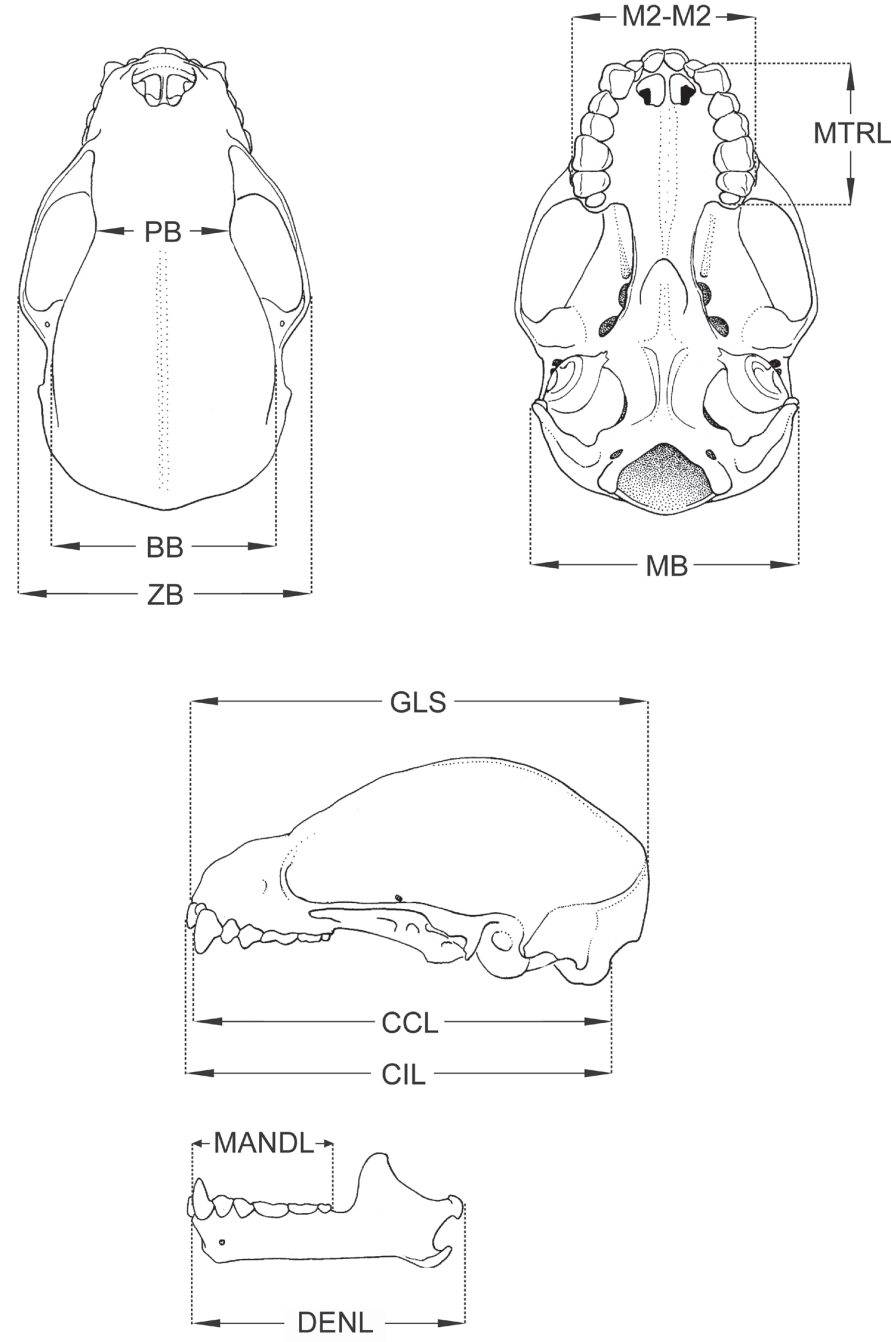
Dorsal and ventral views of the cranium and lateral view of the cranium and mandible illustrating the measurements used in the description. For definitions of abbreviations see Methods.

We examined 62 adult specimens of *Sturnira* representing 8 species of *Sturnira*: 3 specimens of *Sturnira bakeri*, 8 of *Sturnira hondurensis*, 3 of *Sturnira burtonlimi*, 3 of *Sturnira ludovici*, 8 of *Sturnira luisi*, 7 of *Sturnira mordax*, 11 of *Sturnira oporaphilum*, and 19 of *Sturnira parvidens* (see Appendix for complete specimen data). All linear measurements are given in millimeters (mm), weights in grams (g). Standard external measurements (total length, hind foot length, ear length) are those recorded on the specimen labels. We used digital calipers to take one external and 11 craniodental measurements to the nearest 0.01 mm on each specimen (Figure 1). Descriptive statistics (mean and observed range) were calculated for all samples. The craniodental, mandibular, and external measurements used in this study were:

Forearm length (FA): Distance from the elbow (tip of the olecranon process) to the wrist (including the carpals). This measurement was made with the wing at least partially folded.

Greatest length of skull (GLS): Distance from the posteriormost point on the occiput to the anteriormost point on the premaxilla (excluding the incisors).

Condyloincisive length (CIL): Distance between a line connecting the posteriormost margins of the occipital condyles and the anteriormost point on the upper incisors.

Condylocanine length (CCL): Distance between a line connecting the posteriormost margins of the occipital condyles and a line connecting the anteriormost surface of the upper canines.

Postorbital breadth (PB): Least breadth at the postorbital constriction.

Braincase breadth (BB): Greatest breadth of the globular part of the braincase, excluding mastoid and paraoccipital processes.

Mastoid breadth (MB): Greatest breadth across the mastoid region.

Zygomatic breadth (ZB): Greatest breadth across the zygomatic arches.

Maxillary toothrow length (MTRL): Distance from the anteriormost surface of the upper canine to the posteriormost surface of the crown of M3.

Width at M2 (M2–M2): Greatest width of palate across labial margins of the alveoli of M2s.

Dentary length (DENL): Distance from midpoint of condyle to the anteriormost point of the dentary.

Mandibular toothrow length (MANDL): Distance from the anteriormost surface of the lower canine to the posteriormost surface of m3.

## Taxonomy

### Family Phyllostomidae Gray, 1825
Subfamily Stenodermatinae Gervais, 1856
Genus *Sturnira* Gray, 1842

#### Sturnira bakeri sp. n.

http://zoobank.org/1F5CCFAE-60C4-41F4-B0E9-904683866863

http://species-id.net/wiki/Sturnira_bakeri

Baker’s Yellow-shouldered Bat

##### Synonymy.

*Sturnira lilium*: Carrera et al. 2010: 18 (part)

*Sturnira luisi*: Carrera et al. 2010: 18 (part)

*S*[*turnira*]. new species 2: Velazco and Patterson 2013: 687

##### Holotype.

Adult female, deposited at the Museo de Zoología of the Pontificia Universidad Católica del Ecuador (QCAZ 14635), collected on 16 July 2004 by J. Sebastián Tello (original field number JST 487). The body is preserved in alcohol with the skull removed and cleaned. The body and skull are in good condition. Frozen tissues are deposited at Texas Tech University (TK 135127).

##### Type locality.

**Figure 2.**
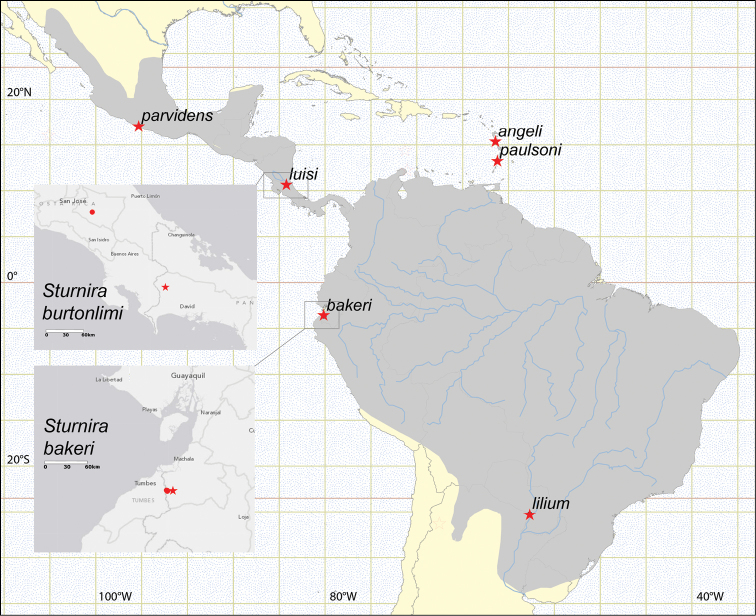
Map of Central and South America, showing the combined distribution range of species formerly ascribed to *Sturnira lilium* (gray tone) and the type localities (stars) of species in the *Sturnira lilium* complex. The localities where *Sturnira burtonlimi* (top inset) and *Sturnira bakeri* (bottom inset) occur are also shown; type localities are represented by a star and paratypes with circles. Note that *Sturnira burtonlimi* and *Sturnira luisi* occur in sympatry.

Palmales, Reserva Militar Arenillas, El Oro, Ecuador, approximately 3°40'27.4"S, 80°06'20"W, 49 m (Figure 2).

##### Paratypes.

An adult female (QCAZ 9737) caught by Peter A. Larsen (original field number PAL 92) and an adult male (QCAZ 9739) caught by Peter A. Larsen (original field number PAL 93), both collected on 16 July 2004 at Quebrada Seca, Fuerte Militar Arenillas (7.1 km W and 12.5 km S of the Military Base), El Oro, Ecuador, approximately 3°39'24.1"S, 80°10'56.2"W, 43 m (Figure 2). Both paratypes are preserved in alcohol. Frozen tissues are deposited at Texas Tech University (TK 135040 and TK 135051 respectively).

##### Distribution.

*Sturnira bakeri* is known from only the two localities in southwestern Ecuador represented by the hypodigm (Figure 2, Appendix). Their proximity to the Peruvian border opens the possibility that *Sturnira bakeri* is also present in northwestern Peru.

##### Etymology.

The name *bakeri* honors our friend Dr. Robert J. Baker, who has made enormous contributions to our understanding of bats, particularly to the evolution of Neotropical phyllostomids. Robert’s numerous contributions, both to the literature and to scientific discourse, and his productive and generous mentoring of students make him a professional paragon for each of us.

##### Measurements.

External and craniodental measurements are presented in Table 1.

**Table 1.** Measurements (mm) and weights (g) of the type series of *Sturnira bakeri* and *Sturnira burtonlimi*.

	*Sturnira bakeri*			*Sturnira burtonlimi*	
	Holotype QCAZ 14635 ♀	Paratype QCAZ 9737 ♀	Paratype QCAZ 9739 ♂	Holotype ROM 104294 ♂	Paratype ROM 104295 ♂
Greatest length of skull	22.7	–	–	22.7	22.8
Condyloincisive length	21.1	–	–	21.5	20.8
Condylocanine length	20.3	–	–	20.6	20.0
Braincase breadth	10.4	–	–	10.5	10.4
Zygomatic breadth	13.5	–	–	13.6	13.5
Postorbital breadth	5.9	–	–	6.5	6.3
Mastoid breadth	11.9	–	–	12.1	11.8
Maxillary toothrow length	6.9	–	–	6.8	6.6
Width at M2	8.3	–	–	8.2	8.1
Dentary length	15.0	–	–	14.9	14.6
Mandibular toothrow length	7.7	–	–	7.6	7.4
Forearm length	45.0	44.0	43.0	44.0	44.0
Total length	65	64	63	72	70
Hind foot length	14	12	14	14	14
Ear length	14	17	15	15	15
Weight	18.7	19.0	21.0	19.0	19.0

##### Diagnosis and description.

**Figure 3.**
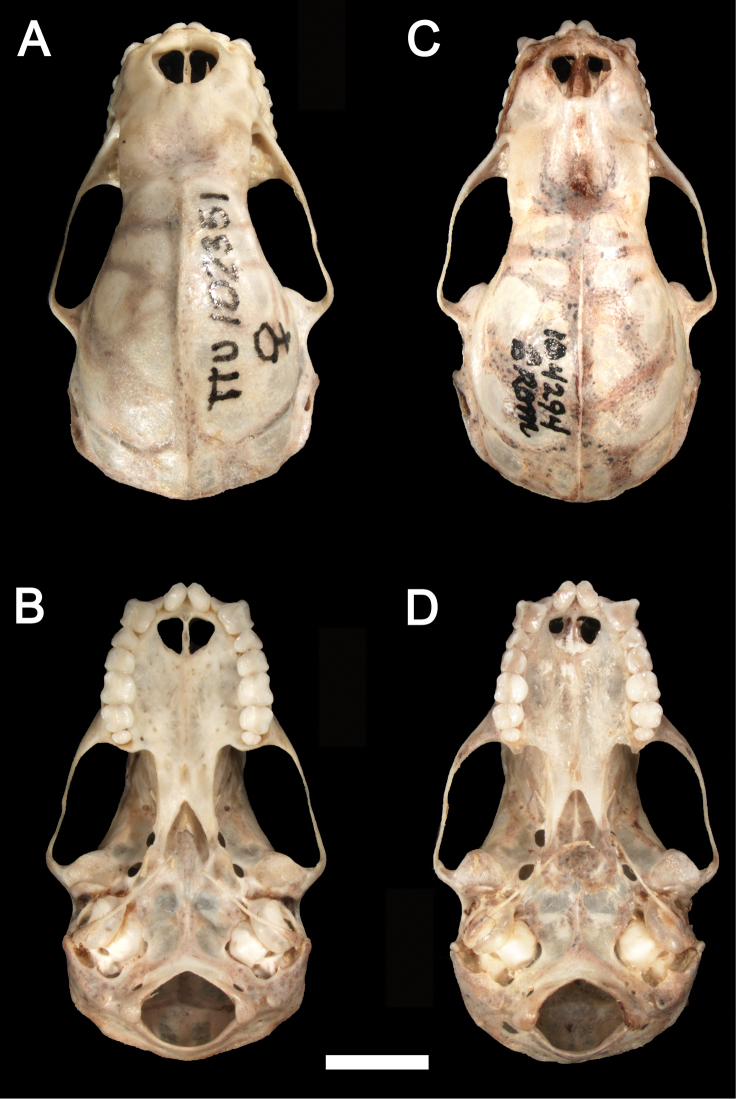
Dorsal (**A**) and ventral (**B**) views of the cranium of *Sturnira bakeri* (QCAZ 14635 ♀) from El Oro, Ecuador. Dorsal (**C**) and ventral (**D**) views of the cranium of *Sturnira burtonlimi* (ROM 104294 ♂) from Chiriquí, Panama. Scale bar = 5 mm.

**Figure 4.**
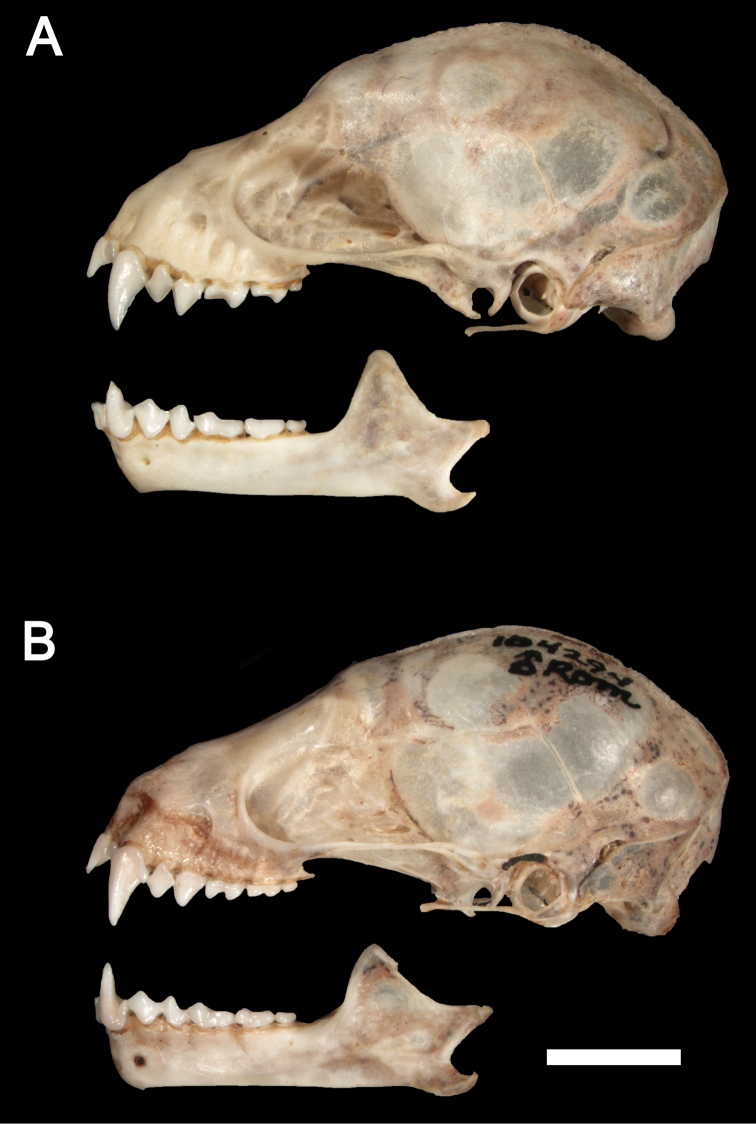
Lateral views of the cranium and mandible of **A** *Sturnira bakeri* (QCAZ 14635 ♀). Lateral views of the cranium and mandible of **B** *Sturnira burtonlimi* (ROM 104294 ♂). Scale bar = 5 mm.

*Sturnira bakeri* is a medium-size yellow-shouldered bat (FA 43.0–45.0 mm; GLS 22.7 mm; CIL 21.1 mm; Table 1) with a slender rostrum and a globular braincase (Figures 3–4). The dorsal fur is pale brown. Dorsal hairs are tetracolored with a short, whitish base (approximately 10% of the length of each hair), a long, pale brown band (approximately 40% of each hair), a long, pale gray band (approximately 40% of each hair), and a short dark brown terminal band (approximately 10% of each hair). The ventral fur is pale gray. Ventral hairs are tricolored with a short, pale gray base (approximately 10% of each hair), a long, pale brown subterminal band (approximately 45% of each hair), and a long, pale gray terminal band (approximately 45% of each hair). The fur is short and woolly, approximately 5 mm long between the shoulders and 5 mm on the chest. The proximal portion of the forearm (roughly 50% of the shaft just distal to the elbow) is sparsely furred with short hairs. The wing membranes are dark brown. The dorsal surfaces of the femur, tibia, and feet are densely covered with long hairs. The IV metacarpal is shorter than the III metacarpal.

**Figure 5.**
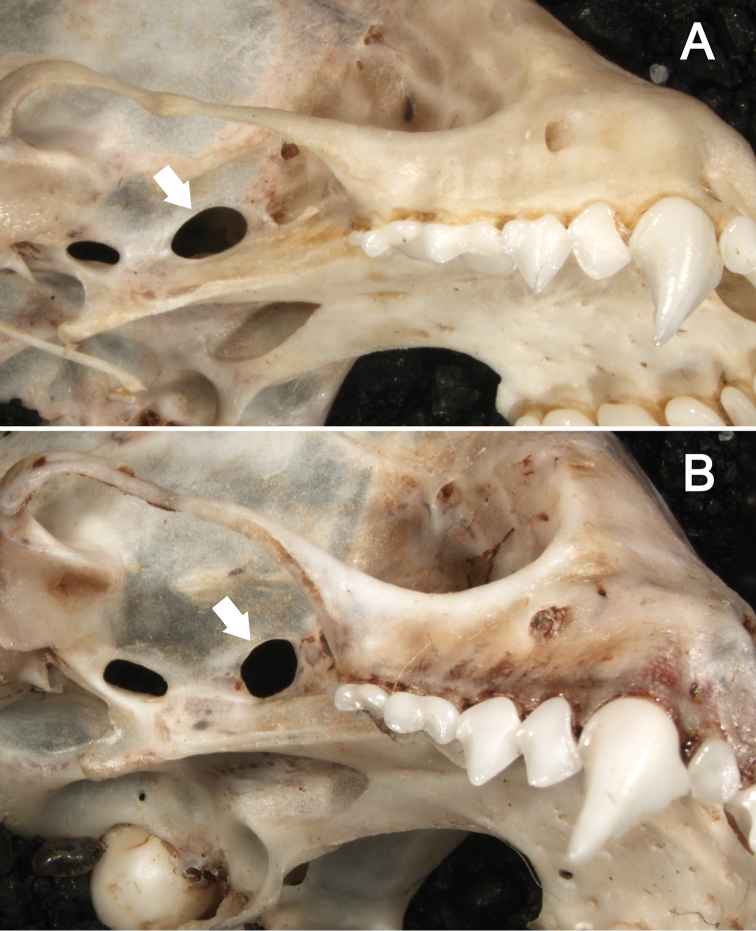
Ventrolateral views of the right orbital region in *Sturnira bakeri* (**A**, QCAZ 14635 ♀) and *Sturnira luisi* (**B**, ROM 104204 ♂) illustrating taxonomic differences in the shape of the sphenorbital fissure. In *Sturnira bakeri*, the sphenorbital fissure is oval (arrow). In *Sturnira luisi*, however, the sphenorbital fissure is semicircular (arrow).

**Figure 6.**
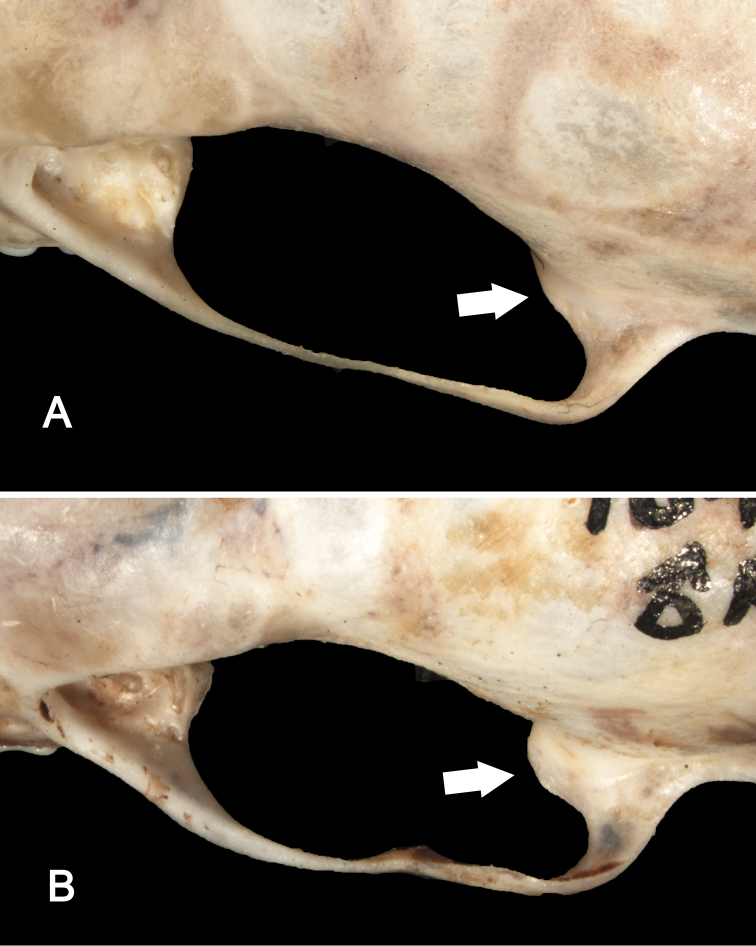
Dorsal view of the left zygomatic arches in *Sturnira bakeri* (**A**, QCAZ 14635 ♀) and *Sturnira luisi* (**B**, ROM 104204 ♂) illustrating taxonomic differences in the development of the glenoid fossa. In *Sturnira bakeri* the anterior process of the glenoid fossa is absent (arrow). In *Sturnira luisi*, however, the anterior process of the glenoid fossa is well developed (arrow).

**Figure 7.**
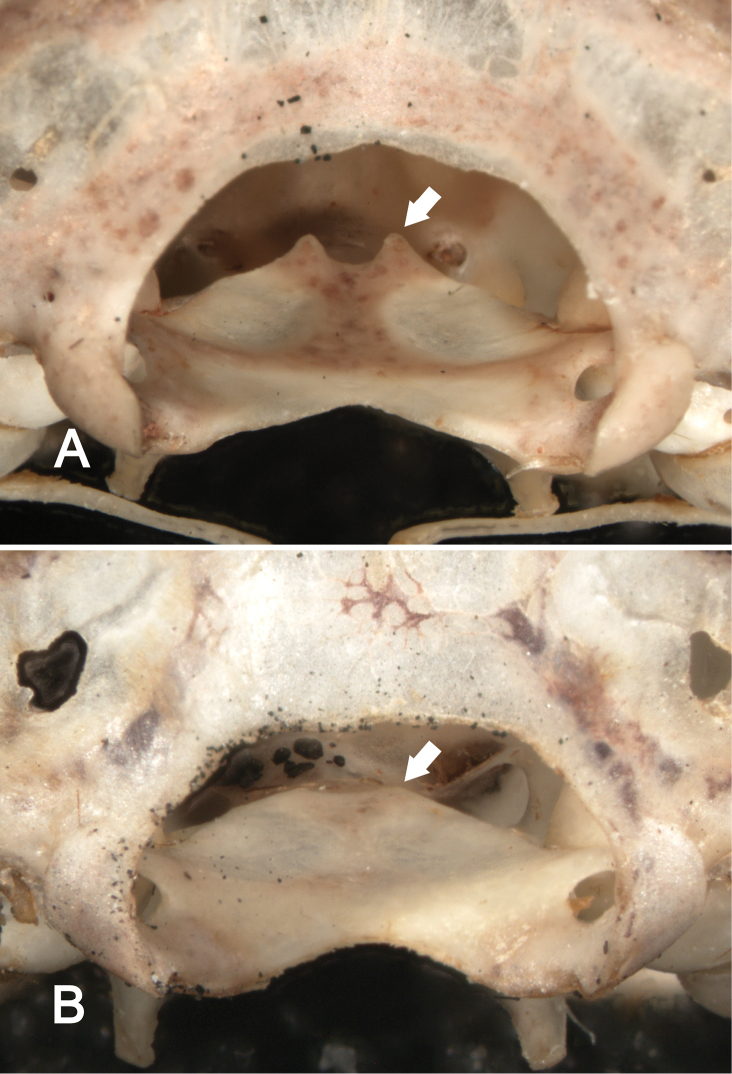
Posterior views of the basioccipital (view through the foramen magnum) in *Sturnira bakeri* (**A**, QCAZ 14635 ♀) and *Sturnira luisi* (**B**, ROM 104204 ♂) illustrating taxonomic differences in the degree of development of the clinoid processes. In *Sturnira bakeri* the clinoid processes are well developed (arrow). In *Sturnira luisi*, however, the clinoid processes are absent (arrow).

The skull of *Sturnira bakeri* has a globular braincase with a slender rostrum and well-developed sagittal crest (Figures 3–4). The basisphenoid pits are shallow and divided by a low midline septum. The sphenorbital fissure is oval (Figure 5). The anterior process of the glenoid fossa is absent (Figure 6). The clinoid processes are well developed (Figure 7), and the proximal end of the stylohyoid is expanded.

**Figure 8.**
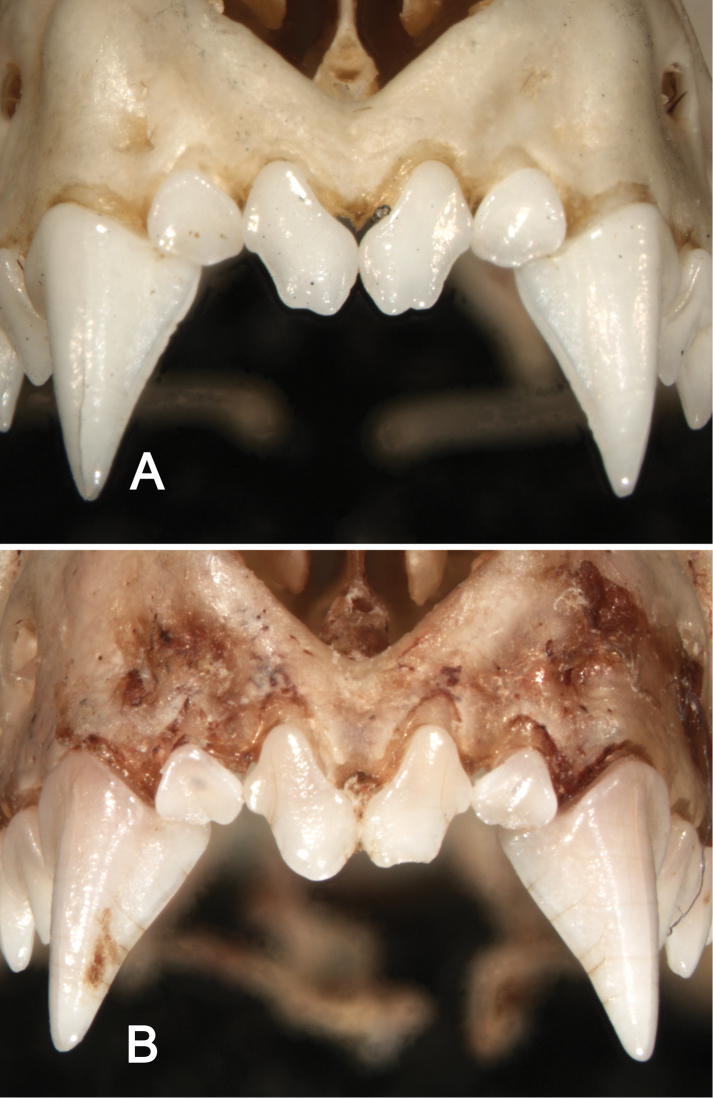
Anterior views of the upper incisors and canines in *Sturnira bakeri* (**A**, QCAZ 14635 ♀) and *Sturnira burtonlimi* (**B**, ROM 104294 ♂) illustrating taxonomic differences in the number of cuspids of the upper inner incisor (I1). In *Sturnira bakeri* the I1 is bicuspidate. In *Sturnira burtonlimi*, however, the I1 is unicuspidate.

**Figure 9.**
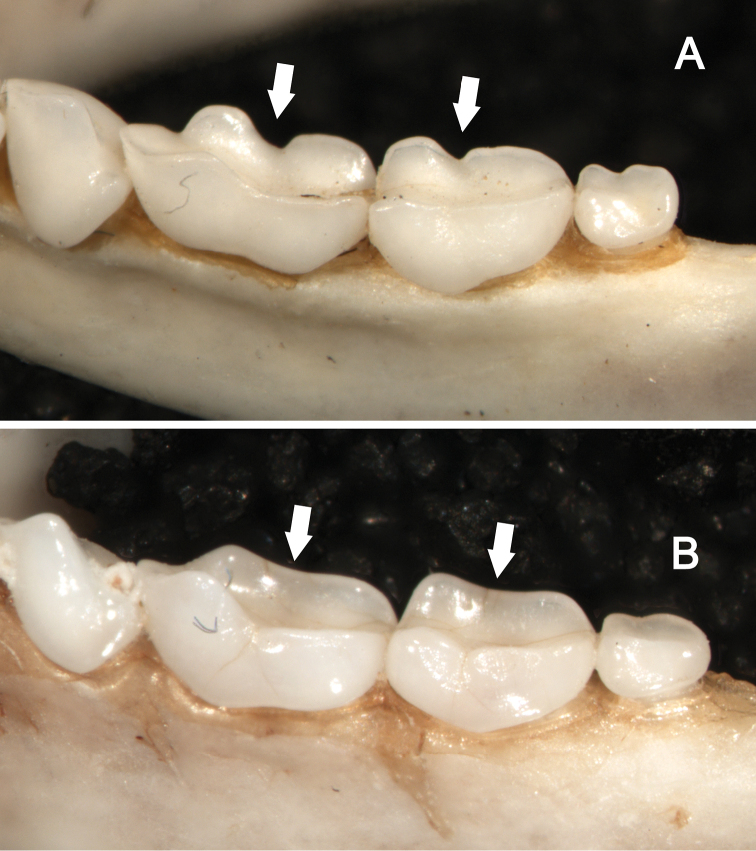
Dorsolateral views of the left mandibular toothrows in *Sturnira bakeri* (**A**, QCAZ 14635 ♀) and *Sturnira burtonlimi* (**B**, ROM 104294 ♂), illustrating taxonomic differences in the shape of the metaconid and entoconid of m1 and m2. In *Sturnira bakeri*, the metaconid and entoconid of m1 and m2 are well defined and separated by a deep notch (arrows). In *Sturnira burtonlimi*, however, the metaconid and entoconid of m1 and m2 are poorly defined and are not separated by a deep notch (arrows).

Like most species of *Sturnira*, *Sturnira bakeri* has a dental formula of I2/2, C1/1, P2/2, M3/3 = 32 teeth. The upper inner incisor (I1) is bicuspidate with a small lateral cusp (Figure 8). The I1 is procumbent and is at least twice the height of the I2. Anteroposterior length of P3 is less than that of P4, and crown height of P3 is slightly less than that of P4. P4 has a small distal cusp. The anteroposterior length of M1 is larger than that of M2. The paracones of M1 and M2 are shorter than their metacones. The direction of the premetacrista of M1 is oblique to the upper alveolar plane. The M3 is ovoid in shape and has two labial cones (cusps). The first and second lower incisors (i1 and i2) are tricuspidate and are subequal in height. The lower canine covers 25% of the occlusal area of i2. The lower canines are laterally divergent, shafts are slanted outward. Anteroposterior length of p2 exceeds that of p4, and crown height of p2 is slightly more than that of p4. The protoconid, hypoconid, metaconid, and entoconid are present in m1 and m2. The paraconid is present and well developed in m1 but absent in m2. The lingual cuspids (metaconid and entoconid) of m1 and m2 are well defined and separated by a deep notch (Figure 9). The m3 is small and only poorly defined, but its protoconid, metaconid, and entoconid are visible.

##### Comparisons.

Comparisons were made with sister species (*Sturnira parvidens*) (Velazco and Patterson 2013) and with other species of the genus (*Sturnira ludovici*, *Sturnira luisi*, and *Sturnira parvidens*) that occur in sympatry with *Sturnira bakeri*. External and craniodental measurements for *Sturnira bakeri* and the compared species are provided in Tables 1 and 2. *Sturnira bakeri* can be easily distinguished from *Sturnira parvidens* by its longer forearm and longer greatest length of skull (Tables 1–2). *Sturnira bakeri*, *Sturnira ludovici*, and *Sturnira luisi* overlap somewhat in size (Tables 1–2) but are unambiguously distinguished by pelage and craniodental characteristics.

**Table 2.** Measurements (mm) of six species of *Sturnira*.

	*Sturnira hondurensis*^a^	*Sturnira ludovici*^b^	*Sturnira luisi*^c^	*Sturnira mordax*^d^	*Sturnira oporaphilum*^e^	*Sturnira parvidens*^f^
FA	45.4 (44.8–46.0) 5	45.6, 45.2, 46.9	46.0, 45.0	44.4, 45.4	47.0 (45.0–48.0) 6	39.9 (38.0–41.0) 12
GLS	23.1 (21.8–24.2) 5	24.4, 23.6, 23.8	23.0, 22.2	21.7, 24.5	23.4 (22.6–24.1) 7	21.2 (20.8–21.8) 9
CIL	21.3 (20.6–22.5) 5	22.7, 22.2, 23.0	–, 20.5	–, 22.5	21.4 (20.9–22.0) 7	19.3 (19.0–19.9) 9
CCL	20.5 (19.6–21.5) 5	21.7, 20.9, 21.7	20.9, 19.7	–, 21.4	20.7 (20.0–21.4) 7	18.6 (18.3–19.2) 9
BB	10.2 (10.1–10.4) 5	10.7, 10.8, 10.5	10.3, 9.9	10.1, 10.5	10.5 (10.5–10.6) 7	10.0 (9.8–10.2) 9
ZB	13.2 (12.5–13.6) 5	14.0, 14.1, 13.5	14.2, 13.7	–, 12.8	13.9 (13.6–14.7) 7	13.0 (12.5–13.4) 9
PB	5.9 (5.7–6.0) 5	6.3, 6.0, 6.0	5.9, 5.9	6.1, 6.1	6.1 (5.8–6.5) 7	5.5 (5.0–5.7) 9
MB	11.8 (11.5–12.0) 5	12.3, 12.1, 12.6	12.0, 11.6	10.6, 11.8	12.2 (11.9–12.4) 7	11.5 (11.2–12.0) 9
MTRL	6.7 (6.5–7.0) 5	7.0, 6.9, 6.9	6.9, 6.8	–, 7.0	6.9 (6.6–7.2) 7	6.2 (6.1–6.4) 9
M2–M2	8.0 (7.5–8.2) 5	8.4, 8.4, 8.3	8.3, 8.1	7.3, 7.5	8.2 (7.8–8.6) 7	7.7 (7.5–8.0) 9
DENL	14.9 (14.4–15.2) 5	15.8, 15.5, 15.5	15.6, 15.0	14.2, 15.5	15.1 (14.8–15.5) 7	13.7 (13.5–14.0) 9
MANDL	7.4 (7.2–7.8) 5	7.9, 7.8, 7.8	7.8, 7.8	7.2, 7.9	7.9 (7.4–8.5) 7	7.1 (6.7–7.4) 9

^a^ Summary statistics (mean, observed range in parentheses, and sample size) for measurements of AMNH 126811 (Holotype,♀); ROM 101366 ♀, ROM 101474 ♀; TTU 83675 ♀, TTU 104945 ♀.

^b^ Measurements of AMNH 67328 (Holotype, ♂); TTU 102457 ♀, TTU 102461 ♂.

^c^ Measurements of ROM 104204 ♂, ROM 105807 ♂.

^d^ Measurements of AMNH 142485 ♀; FMNH 124092 ♂.

^e^ Summary statistics (mean, observed range in parentheses, and sample size) for measurements of AMNH 263462 ♀, AMNH 263463 ♀, AMNH 263465 ♂, AMNH 264660 ♂; FMNH 128926 ♀, FMNH 174843 ♂; MUSM 39230 ♂.

^f^ Summary statistics (mean, observed range in parentheses, and sample size) for measurements of ROM 96276 ♀, ROM 97412 ♀, ROM 99284 ♂; TTU 44789 ♀, TTU 61103 ♂, TTU 62410 ♀, TTU 62411 ♂, TTU 84422 ♀, TTU 84608 ♀, TTU 104285 ♀, TTU 104631 ♀, TTU 105076 ♂.

Externally, the dorsal pelage between the shoulders of *Sturnira bakeri*, *Sturnira luisi*, and *Sturnira parvidens* is short (4.0–6.0 mm) whereas it is long (10 mm) in *Sturnira ludovici*. Individual dorsal hairs are tetracolored in *Sturnira bakeri* and *Sturnira ludovici* whereas they are bicolored in *Sturnira luisi* and *Sturnira parvidens*. The overall appearance of the dorsal pelage is pale brown in *Sturnira bakeri* and *Sturnira luisi*, whereas it is dark brown in *Sturnira ludovici* and reddish in *Sturnira parvidens*. Ventrally the hairs are short (4.0–6.0 mm) and tricolored in *Sturnira bakeri*, *Sturnira ludovici*, and *Sturnira parvidens*, but short (4.0–6.0 mm) and bicolored in *Sturnira luisi*. The ventral fur is pale gray in *Sturnira bakeri*, whereas it is dark gray in *Sturnira luisi*, dark brown in *Sturnira ludovici*, and reddish in *Sturnira parvidens*. Shoulder glands (epaulettes) are conspicuous in *Sturnira bakeri*, *Sturnira ludovici*, *Sturnira luisi*, and *Sturnira parvidens*. The trailing edge of the uropatagium is covered by short hairs (4.0–5.0 mm) in *Sturnira bakeri*, *Sturnira luisi*, and *Sturnira parvidens*, whereas the uropatagium is covered by long hairs (7.0–9.0 mm) in *Sturnira ludovici*. The proximal portion of the forearm (roughly 50% of the shaft just distal to the elbow) is sparsely furred with short hairs in *Sturnira bakeri* and *Sturnira luisi*, whereas it is well furred with short hair in *Sturnira ludovici* and *Sturnira parvidens*. The dorsal surfaces of the femur and tibia are densely covered with long hairs in *Sturnira bakeri*, whereas they are densely covered with short hairs in *Sturnira ludovici*, sparsely covered with long hairs in *Sturnira parvidens*, and sparsely covered with short hairs in *Sturnira luisi*. The dorsal surfaces of the feet are densely covered with long hairs in *Sturnira bakeri* and *Sturnira parvidens*, whereas they are densely covered with short hairs in *Sturnira ludovici* or sparsely covered with short hairs in *Sturnira luisi*. The IV metacarpal is shorter than the III metacarpal in *Sturnira bakeri* and *Sturnira parvidens*, whereas the IV metacarpal is equal to the III metacarpal in *Sturnira ludovici* and *Sturnira luisi*.

Cranially, the rostrum of *Sturnira bakeri*, *Sturnira ludovici*, and *Sturnira luisi* is slender, whereas it is broad in *Sturnira parvidens*. The zygomatic arches are straight in *Sturnira bakeri* and *Sturnira luisi*, whereas they are bowed outward in *Sturnira ludovici* and *Sturnira parvidens*. The basisphenoid pits are shallow and divided by a low midline septum in *Sturnira bakeri* and *Sturnira ludovici*, whereas they are shallow and divided by a high septum in *Sturnira luisi* and deep and divided by a high septum in *Sturnira parvidens*. The sphenorbital fissure is oval in *Sturnira bakeri*, *Sturnira ludovici*, and *Sturnira parvidens*, whereas it is subcircular in *Sturnira luisi* (Figure 5). The anterior process of the glenoid fossa is absent in *Sturnira bakeri*, whereas it is well developed in *Sturnira luisi* and *Sturnira parvidens* (Figure 6). Some specimens of *Sturnira ludovici* lack the anterior process of the glenoid fossa (TTU 102461) while in others (TTU 102457) it is well developed. The clinoid processes are present and well developed in *Sturnira bakeri* and *Sturnira ludovici*, whereas they are absent in *Sturnira luisi* (Figure 7). Some specimens of *Sturnira parvidens* lack clinoid processes (ROM 97412; TTU 84608) while others (ROM 99284) possess them. The proximal end of the stylohyoid is expanded in *Sturnira bakeri*, *Sturnira ludovici*, and *Sturnira parvidens*, whereas it is narrow in *Sturnira luisi*.

Dentally, two labial cusps are present in M3 in *Sturnira bakeri*, *Sturnira luisi* and *Sturnira parvidens*, whereas only one labial cusp is present *Sturnira ludovici*. The i1 and i2 are tricuspidate in *Sturnira bakeri*, *Sturnira luisi*, and *Sturnira parvidens*, whereas they are bicuspidate in *Sturnira ludovici*. The metaconid and entoconid of m1 and m2 are well defined and separated by a deep notch in *Sturnira bakeri*, *Sturnira luisi*, and *Sturnira parvidens*, whereas they are poorly defined and are not separated by a deep notch in *Sturnira ludovici* (Figure 9).

##### Natural history.

The area surrounding the Fuerte Militar Arenillas is relatively xeric and surrounded by primary dry forest, secondary forest, and plantations of crops (Carrera et al. 2010). No other information is available.

#### Sturnira burtonlimi sp. n.

http://zoobank.org/309E2CD7-7E93-4E46-89A9-C6EF685FE5C5

http://species-id.net/wiki/Sturnira_burtonlimi

Burton’s Yellow-shouldered Bat

##### Synonymy.

*Sturnira ludovici*: Clare et al. 2011: 9 (part)

*S*[*turnira*]. new species 1: Velazco and Patterson 2013: 687

##### Holotype.

Adult male, deposited at the Royal Ontario Museum (ROM 104294), collected on 7 March 1995 by Burton K. Lim and Eamon O’Toole (original field number F 38144). Prepared as dry skin, skull, and skeleton. The skin, skull, and skeleton are in good condition. Frozen tissues are deposited at the Royal Ontario Museum (F 38144).

##### Type locality.

Ojo de Agua, 2 km N of Santa Clara, Chiriquí, Panama, approximately 8°42'N, 82°45'W, 1500m (Figure 2).

**Paratype.** The skin, skull, and carcass of an adult male (ROM 104295) caught on 7 March 1995 at the type locality by Burton K. Lim and Eamon O’Toole (original field number F 38145).

##### Additional specimens.

Besides the specimens from the type series from Panama, Velazco and Patterson (2013) reported an additional record of *Sturnira burtonlimi* (referred as *Sturnira* new species 1) from the Cartago province in Costa Rica (MVZ 174432 ♂; Appendix) based on DNA sequence data. We did not include this specimen as part of the type series because it was not available for examination, therefore the diagnostic morphological characteristics of *Sturnira burtonlimi* could not be confirmed in this specimen.

##### Distribution.

The new species is known from only two localities, one in Costa Rica and the other in Panama (Figure 2, Appendix).

##### Etymology.

The name *burtonlimi* honors our friend Dr. Burton K. Lim, who collected the type series of this species and has made many other important collections throughout the Neotropics and beyond. Burton is a tireless fieldworker whose research has contributed much to our understanding of the diversity, relationships, and biogeography of tropical mammals.

##### Measurements.

External and craniodental measurements are presented in Table 1.

##### Diagnosis and description.

*Sturnira burtonlimi* is a medium-sized yellow-shouldered bat (FA 44.0 mm; GLS 22.7–22.8 mm; CIL 20.8–21.5 mm; Table 1) with a broad rostrum and a globular braincase (Figures 3–4). The dorsal fur is dark brown. Dorsal hairs are tetracolored with a short, pale gray base (approximately 10% of the length of each hair), a long, dark grey band (approximately 40% of each hair), a long, pale gray band (approximately 40% of each hair), and short dark brown terminal band (approximately 10% of each hair). The ventral fur is dark brown. Ventral hairs are tricolored with a short, pale gray base (approximately 10% of each hair), a long, dark brown subterminal band (approximately 45% of each hair), and a long, gray terminal band (approximately 45% of each hair). The fur is long and woolly, approximately 7 mm long between the shoulders and 5 mm on the chest. The proximal portion of the forearm (roughly 50% of the shaft just distal to the elbow) is densely furred with short hairs. The wing membranes of *Sturnira burtonlimi* are dark brown. The dorsal surfaces of the femur and tibia are densely covered with long hairs. The dorsal surfaces of the feet are densely covered with short hairs. The IV metacarpal is shorter than the III metacarpal.

The skull of *Sturnira burtonlimi* has a globular braincase with a broad rostrum and a well-developed sagittal crest (Figures 3–4). The basisphenoid pits are shallow and divided by a low midline septum. The sphenorbital fissure is subcircular. The anterior process of the glenoid fossa is well developed, as are the clinoid processes. The proximal end of the stylohyoid is expanded.

Like most species of *Sturnira*, *Sturnira burtonlimi* has a dental formula of I2/2, C1/1, P2/2, M3/3 = 32 teeth. The upper inner incisor (I1) is unicuspidate and has a small lateral cusp (Figure 8). The I1 is procumbent and is at least twice the height of the I2. Anteroposterior length of P3 is less than that of P4, and crown height of P3 is slightly less than that of P4. Both P3 and P4 possess a small distal cusp. Anteroposterior length of M1 is longer than M2. The paracone and metacone of M1 and M2 are subequal in height. The direction of the premetacrista of M1 is perpendicular to the upper alveolar plane. The M3 is ovoid in shape and has only one labial cone (cusp). The first and second lower incisors (i1 and i2) are bicuspidate. The i1 and i2 are subequal in height. The lower canine covers half the occlusal surface of i2. The lower canines are laterally divergent, their shafts slanted outward. Anteroposterior length of p2 is more than that of p4, and crown height of p2 is slightly more than that of p4. The protoconid, hypoconid, metaconid, and entoconid are present in m1 and m2. The paraconid is present and well developed in m1. Paraconid is absent in m2. The lingual cuspids (metaconid and entoconid) of m1 and m2 are poorly defined and are not separated by a deep notch (Figure 9). The m3 is small and only poorly defined protoconid, metaconid, and entoconid are evident.

##### Comparisons.

*Sturnira burtonlimi* was compared with the closely related species *Sturnira hondurensis*, *Sturnira ludovici*, and *Sturnira oporaphilum* (Velazco and Patterson 2013) and with other sympatric species of the genus (*Sturnira luisi*, *Sturnira mordax*, and *Sturnira parvidens*). External and craniodental measurements for *Sturnira burtonlimi* and the compared species are provided in Tables 1 and 2. *Sturnira burtonlimi* can be easily distinguished from *Sturnira ludovici* by its shorter forearm and shorter greatest length of skull and from *Sturnira parvidens* by its longer forearm and longer greatest length of skull (Tables 1–2). *Sturnira burtonlimi*, *Sturnira hondurensis*, *Sturnira luisi*, *Sturnira mordax*, and *Sturnira oporaphilum* overlap somewhat in size (Tables 1–2) but can be unambiguously distinguished based on pelage and craniodental characteristics.

Externally, the dorsal pelage between the shoulders of *Sturnira burtonlimi*, *Sturnira hondurensis*, *Sturnira ludovici*, *Sturnira mordax*, and *Sturnira oporaphilum* is long (7.0–10 mm) and tetracolored, whereas it is short (4.0–6.0 mm) and bicolored in *Sturnira luisi* and *Sturnira parvidens*. The overall appearance of the dorsal pelage is dark brown in *Sturnira burtonlimi*, *Sturnira hondurensis*, *Sturnira ludovici*, *Sturnira mordax*, and *Sturnira oporaphilum*, whereas it is pale brown in *Sturnira luisi* and reddish in *Sturnira parvidens*. Ventrally the hairs are short (4.0–6.0 mm) and tricolored in *Sturnira burtonlimi*, *Sturnira ludovici*, *Sturnira mordax*, *Sturnira oporaphilum*, and *Sturnira parvidens*; but short (4.0–6.0 mm) and bicolored in *Sturnira luisi*, and long (8.0 mm) and monocolored in *Sturnira hondurensis*. The ventral fur is dark gray in *Sturnira burtonlimi*, *Sturnira luisi*, and *Sturnira oporaphilum*, whereas it is pale gray in *Sturnira hondurensis*, dark brown in *Sturnira ludovici*, *Sturnira mordax*, and reddish in *Sturnira parvidens*. Shoulder glands (epaulettes) are conspicuous in *Sturnira burtonlimi*, *Sturnira ludovici*, *Sturnira luisi*, and *Sturnira parvidens*, whereas they are absent in *Sturnira hondurensis*, *Sturnira mordax*, and *Sturnira oporaphilum*. The trailing edge of the uropatagium is covered by long hairs (7.0–9.0 mm) in *Sturnira burtonlimi*, *Sturnira hondurensis*, *Sturnira ludovici*, and *Sturnira oporaphilum*, whereas the uropatagium is covered by short hairs (4.0–5.0 mm) in *Sturnira luisi*, *Sturnira mordax*, and *Sturnira parvidens*. The proximal portion of the forearm (roughly 50% of the shaft just distal to the elbow) is well furred with short hair in *Sturnira burtonlimi*, *Sturnira ludovici*, and *Sturnira parvidens*, whereas it is well furred with long hair in *Sturnira hondurensis*, *Sturnira mordax*, and *Sturnira oporaphilum*, and sparsely furred with short hairs in *Sturnira luisi*. The dorsal surfaces of the femur and tibia are densely covered with long hairs in *Sturnira burtonlimi* and *Sturnira hondurensis*, whereas they are densely covered with short hairs in *Sturnira ludovici*, sparsely covered with long hairs in *Sturnira mordax*, *Sturnira oporaphilum*, and *Sturnira parvidens*, and sparsely covered with short hairs in *Sturnira luisi*. The dorsal surfaces of the feet are densely covered with short hairs in *Sturnira burtonlimi* and *Sturnira ludovici*, whereas they are densely covered with long hairs in *Sturnira hondurensis*, *Sturnira oporaphilum*, and *Sturnira parvidens*, sparsely covered with long hairs in *Sturnira mordax*, and sparsely covered with short hairs in *Sturnira luisi*. The IV metacarpal is shorter than the III metacarpal in *Sturnira burtonlimi*, *Sturnira hondurensis*, and *Sturnira parvidens*, whereas the IV metacarpal is equal to the III metacarpal in *Sturnira ludovici*, *Sturnira luisi*, *Sturnira mordax*, and *Sturnira oporaphilum*.

Cranially, the rostrum of *Sturnira burtonlimi*, *Sturnira oporaphilum*, and *Sturnira parvidens* is broad, whereas it is slender in *Sturnira hondurensis*, *Sturnira ludovici*, *Sturnira luisi*, and *Sturnira mordax*. The basisphenoid pits are shallow and divided by a low midline septum in *Sturnira burtonlimi*, *Sturnira ludovici*, *Sturnira mordax*, and *Sturnira oporaphilum*, whereas they are shallow divided by a high septum in *Sturnira luisi* and deep divided by a high septum in *Sturnira hondurensis* and *Sturnira parvidens* (Figure 3). The sphenorbital fissure is subcircular in *Sturnira burtonlimi*, *Sturnira hondurensis*, *Sturnira luisi*, *Sturnira mordax*, and *Sturnira oporaphilum*, whereas it is oval in *Sturnira ludovici* and *Sturnira parvidens*. The anterior process of the glenoid fossa is well developed in *Sturnira burtonlimi*, *Sturnira hondurensis*, *Sturnira luisi*, and *Sturnira parvidens*, whereas it is absent or poorly developed in *Sturnira mordax* and *Sturnira oporaphilum*. Some specimens of *Sturnira ludovici* (TTU 102461) lack the anterior process of the glenoid fossa while others (TTU 102457) present a well-developed anterior process of the glenoid fossa. The clinoid processes are well developed in *Sturnira burtonlimi* and *Sturnira ludovici*, whereas they are weak in *Sturnira oporaphilum* and absent in *Sturnira hondurensis*, *Sturnira luisi*, and *Sturnira mordax*. Clinoid processes are present in some specimens of *Sturnira parvidens* (ROM 99284), while they are lacking in others (ROM 97412; TTU 84608). The proximal end of the stylohyoid is expanded in *Sturnira burtonlimi*, *Sturnira ludovici*, *Sturnira oporaphilum*, and *Sturnira parvidens*, whereas it is narrow in *Sturnira hondurensis*, *Sturnira luisi*, and *Sturnira mordax*.

Dentally, the upper inner incisor (I1) is unicuspidate in *Sturnira burtonlimi* and *Sturnira hondurensis*, whereas it is bicuspidate in *Sturnira ludovici*, *Sturnira luisi*, *Sturnira mordax*, *Sturnira oporaphilum*, and *Sturnira parvidens* (Figure 8). A small distal cusp is present on P3 in *Sturnira burtonlimi* and *Sturnira oporaphilum*, whereas this cusp is absent in *Sturnira hondurensis*, *Sturnira ludovici*, *Sturnira luisi*, *Sturnira mordax*, and *Sturnira parvidens*. The direction of the premetacrista of M1 is perpendicular to the upper alveolar plane in *Sturnira burtonlimi*, whereas the premetacrista is oblique to the upper alveolar plane in *Sturnira hondurensis*, *Sturnira ludovici*, *Sturnira luisi*, *Sturnira mordax*, *Sturnira oporaphilum*, and *Sturnira parvidens*. One labial cusp is present in M3 in *Sturnira burtonlimi*, *Sturnira hondurensis*, *Sturnira ludovici*, and *Sturnira oporaphilum*, whereas two labial cusps are present in *Sturnira luisi* and *Sturnira parvidens*. The i1 and i2 are bicuspidate in *Sturnira burtonlimi*, *Sturnira hondurensis*, *Sturnira ludovici*, *Sturnira mordax*, and *Sturnira oporaphilum*, whereas they are tricuspidate in *Sturnira luisi* and *Sturnira parvidens*. The lower canines are laterally divergent, shafts slanted outward, in *Sturnira burtonlimi*, *Sturnira ludovici*, *Sturnira luisi*, and *Sturnira parvidens*, whereas they are not laterally divergent in *Sturnira hondurensis*, *Sturnira mordax*, and *Sturnira oporaphilum*. The metaconid and entoconid of m1 and m2 are poorly defined and are not separated by a deep notch in *Sturnira burtonlimi*, *Sturnira hondurensis*, *Sturnira ludovici*, *Sturnira mordax*, and *Sturnira oporaphilum*, whereas the metaconid and entoconid of m1 and m2 are well defined and separated by a deep notch in *Sturnira luisi* and *Sturnira parvidens* (Figure 9).

##### Natural history.

*Sturnira burtonlimi* has been documented from an elevational range of 1290 to 1500 m and was taken in premontane forest near coffee fields. All known specimens are males. Testes of the type series specimens measured 5 × 3 mm (ROM 104294) and 7 × 5 mm (ROM 104295).

## Discussion

From the time of its description in 1810, *Sturnira lilium* was thought to be one of the most widespread species of phyllostomid bats, ranging from northern Mexico to northern Argentina and into the Lesser Antilles. Six subspecies have been recognized (Gannon et al. 1989, Gardner 2008): *Sturnira lilium lilium* Geoffroy St.-Hilaire, 1810 over much of South America east of the Andes; *Sturnira lilium parvidens* Goldman, 1917 over much of Central America and Pacific slopes of Colombia and Ecuador; and four subspecies restricted to the Lesser Antilles: *Sturnira lilium angeli* de la Torre, 1966; *Sturnira lilium luciae* Jones & Phillips, 1976; *Sturnira lilium paulsoni* de la Torre & Schwartz, 1966; and *Sturnira lilium zygomaticus* Jones & Phillips, 1976. Multilocus molecular analyses of the genus (Velazco and Patterson 2013) showed *Sturnira lilium* to be a paraphyletic complex of six species, including two that lacked names. Their analyses suggested that four of the six erstwhile subspecies of *Sturnira lilium* should be elevated to specific rank, namely *Sturnira angeli*, *Sturnira lilium*, *Sturnira parvidens*, and *Sturnira paulsoni*. The other two subspecies were considered junior synonyms: *zygomaticus* of *Sturnira angeli* (which was also shown to include “*Sturnira thomasi*”); and *luciae* of *Sturnira paulsoni* (Figure 2).

With the descriptions of two additional species in the *lilium* complex (*Sturnira bakeri* in this report and “*Sturnira* new species 3” of Velazco and Patterson (2013), to be described elsewhere), the distribution of *Sturnira lilium* has been radically altered. *Sturnira lilium* is actually restricted to the Brazilian Shield portions of Brazil, Bolivia, Paraguay, and Argentina. However, most reports on the ecology, distribution, morphology, behavior, and parasites of *Sturnira lilium* are based on different species (e.g., Wenzel and Tipton 1966, Wenzel 1976, Contreras Vega and Cadena 2000, Evelyn and Stiles 2003, Lobova et al. 2009, Medina and Lopez 2010, Jarrín-V and Kunz 2011, Jarrín-V and Clare 2013, Frank et al. 2014, Melaun et al. 2014). The largely allopatric distributions of forms in this species complex should aid efforts to allocate these observations to the correct species.

In a similar manner, Velazco and Patterson (2013) found that *Sturnira ludovici* and *Sturnira oporaphilum* were sister species and related to two unrecognized species. Their sister species is here named *Sturnira burtonlimi*. All three species are then sister to a Central American taxon long considered a subspecies of *Sturnira ludovici* (Simmons 2005), which should be recognized as *Sturnira hondurensis* as suggested by Gardner (2008).

According to the timetree analysis of Velazco and Patterson (2013), these newly described species arose after the final emergence of the Panamanian landbridge during the Pliocene. Both *Sturnira burtonlimi*, a Central American form, and *Sturnira bakeri*, a South American form, have their closest living relative on the opposite side of the Panamanian isthmus: *Sturnira burtonlimi* is sister to *Sturnira ludovici* + *Sturnira oporaphilum*, and *Sturnira bakeri* is sister to *Sturnira parvidens*. Both of these divergence events were dated to the Late Pliocene or Early Pleistocene.

## Supplementary Material

XML Treatment for Sturnira bakeri

XML Treatment for Sturnira burtonlimi

## Appendix

Specimens of *Sturnira* used in this study. See Methods for collection acronyms. Individuals marked with an asterisk were used only in the molecular analyses.

*Sturnira bakeri* new species (3).– ECUADOR: *El Oro*: Reserva Militar Arenillas, Palmales (QCAZ 14635 [holotype]); Fuerte Militar Arenillas (7.1 km W and 12.5 km S of the Military Base), Quebrada Seca (QCAZ 9737, 9739).

*Sturnira burtonlimi* new species (3).– COSTA RICA: *Cartago*: Colima Tapanti, 1.6 km S Tapanti, Bridge over río Grande de Orosi (MVZ 174432*). PANAMA: *Chiriquí*: Ojo de Agua, 2 km N Santa Clara (ROM 104294 [holotype], 104295).

*Sturnira hondurensis* (8).– EL SALVADOR: *Santa Ana*: Los Planes (ROM 101474); Parque Nacional Montecristo, Bosque Nebuloso (ROM 101366). GUATEMALA: *El Progreso*: 3 km W Pinalon, Reserva de Biosfera Sierra de las Minas, Municipalidad San Agustin Acasaguastlan (MVZ 223393*). *Huehuetenango*: 2.5 km S, 2.75 km W San Mateo Ixtatan (MVZ 223172*); Finca Ixcansan, 10.3 km (by road) E of Yalambojoch on road to Rio Seco, Sierra de los Cuchumatanes (MVZ 223178*). HONDURAS: *Francisco Morazán*: La Tigra Parque Nacional (TTU 83675). *La Paz*: La Cruz Grande (AMNH 126811 [holotype]). MEXICO: *San Luis Potosí*: 1.5 mi W Las Abritas (TTU 104945).

*Sturnira ludovici* (3).– ECUADOR: *El Oro*: Jardin Botánico Moro Moro (limite con la Reserva Jocotoco) (TTU 102457, 102461). *Pichincha*: W side Pichincha near Gualea (AMNH 67328 [holotype]).

*Sturnira luisi* (8).– ECUADOR: *Esmeraldas*: 2 km S of Alto Tambo (ROM 105807); terrenos aledaños de La Comuna San Francisco de Bogota (TTU 103217*). PANAMA: *Bocas del Toro*: Isla Popa, S Shore, 1 km E Sumwood Channel (USNM 579052*); Isla San Cristobal, Bocatorito (USNM 449721*); Peninsula Valiente, Bahia Azul, Pigeon Key Trail (USNM 578239*). *Canal Zone*: Gamboa (ROM 104204). *Chiriquí*: Santa Clara (TTU 39136*). *Darién*: Caña (LSUMZ 25478*).

*Sturnira mordax* (7).– COSTA RICA: *Alajuela*: 4.2 km SE Cariblanco (CM 92486–92488*; TJM 6741*). *Cartago*: Refugio Nacional Tapanti, Sombrilla de Pobre Trail at Quebrada Segunda Trail, 0.2 km N park headquarters (MVZ 174439*. *Puntarenas*: Cañas Gordas, Las Vueltas (AMNH 142485); Finca Las Cruces, 2 km S San Vito (FMNH 124092).

*Sturnira oporaphilum* (11).– BOLIVIA: *La Paz*: 3 km S of Irupana (AMNH 263462–263463, 263465); Chijchipa (AMNH 264660). ECUADOR: *Tungurahua*: La Estancia (TTU 84970*). PERU: *Amazonas*: Luya, Río Utcubamba, 11 km by road NW Pedro Ruiz (FMNH 128925*). *Cajamarca*: Chota Querocoto, Monte Ribereño (MUSM 39428*); Santa Cruz, Río Zaña, 2 km N Monte Seco (FMNH 128926). *Cuzco*: Paucartambo, Consuelo, 15.9 km SW Pilcopata (FMNH 174843). *Madre de Dios*: Manu, Maskoitania, 13.4 km NNW Atalaya, left bank Río Alto Madre de Dios (FMNH 174844*). *San Martín*: Moyobamba, Area de Conservación Municipal Mishquiyacu Rumiyacu-Almendra, Orquidiario Waqanki (MUSM 39230).

*Sturnira parvidens* (19).– COSTA RICA: *Puntarenas*: 5 km S, 6 km W Esparza (LSUMZ 28341*). EL SALVADOR: *La Paz*: 1 mi N La Herradura (TTU 62410). *Santa Ana*: Cemetery, 2 mi S Santa Ana (TTU 62411). GUATEMALA: *El Peten*: Tikal (ROM 99284). HONDURAS: *Atlántida*: Lancetilla Botanical Garden (TTU 84422). *Comayagua*: Parque Nacional Cerro Azul, Meambar (TTU 104285). *Copán*: 5 km NW Santa Rosa de Copan (TTU 84608). *Valle*: 8.5 mi SSW San Lorenzo (TTU 61103). MEXICO: *Campeche*: 3.7 km SE of Chekubul (ROM 96276). *Chiapas*: 25 km S, 3 km N Ocozocoautla (TTU 104631). *Quintana Roo*: 6 km S of Majahual (ROM 97412). *Sonora*: 12 mi E (by road) Alamos, Rio Cuchujaqui (MSB 53756*, 53759*, 53760*); 3 mi S, 2.8 mi W Alamos, La Aduana, Santo Domingo (MSB 53758*). *Tamaulipas*: 2 mi W Calabazas, Río Sabinas (TTU 44789). *Veracruz*: La Mancha Field Station (MSB 82216*, 82218*); Paso del Patal [=Panal?] (TTU 105076).
